# Measuring Healthcare Worker Satisfaction in the Operating Room

**DOI:** 10.1097/AS9.0000000000000129

**Published:** 2022-01-21

**Authors:** Alonzo D. Jones, Hunter D. D. Witmer, Çağla Keceli, Mihai Giurcanu, Dan Adelman, Kiran K. Turaga

**Affiliations:** From the * The University of Chicago Pritzker School of Medicine, Chicago, IL; † Department of Surgery, The University of Chicago Medicine, Chicago, IL; ‡ Booth School of Business, The University of Chicago, Chicago, IL; § Department of Public Health Sciences, The University of Chicago, Chicago, IL.

## Abstract

Mini-abstract: This report highlights the efficacy of using a 5-point Likert scale to measure healthcare worker satisfaction in the operating room. This assessment is significant because it is a critical step in assessing a novel scheduling apparatus that hopes to improve team satisfaction, operative efficiency, and operating room waste.

## INTRODUCTION

Lack of satisfaction of an operating room (OR) team can lead to more frequent clinical errors and longer operative times.^1^ The Safety Attitudes Questionnaire (SAQ) is a well described and validated psychometric tool for assessing individual perceptions of teamwork quality in healthcare settings, including the OR.^2–4^ SAQ items assess perceptions across 6 climate domains: teamwork climate, safety climate, job satisfaction, stress recognition, perceptions of management, and working conditions using a 5-point Likert scale.

Our team is taking a novel approach to assessing operative team satisfaction at our institution. We hypothesize that the installation of HappyOrNot devices, a tool commonly used to survey customer satisfaction in commercial venues, will enable us to accurately collect real-time data on case-specific operative team satisfaction. Each device will record responses to a single prompt, “Compared to the typical case in your specialty, how satisfied are you with the teamwork in this case?”. To validate the prompt prior to installation of the devices, a 17-item survey was created that included selected items from the SAQ, which were chosen from the teamwork and patient safety climates and adapted to reference a single operative case for contextual applicability.

## METHODS

Three ORs were randomly selected each morning using a random number table for 11 consecutive business days. All assigned OR staff (surgeons, anesthesiologists, residents/fellows, circulating nurse, and scrub nurse) were emailed a secured research electronic capture survey at 9:00 am to assess perceptions of teamwork following the first case of the day. All responses were anonymous and confidential. The responses were curated for timely completion (<1 wk from the case).

## RESULTS

Of the 194 surveys distributed, 64 were completed and returned within 7 days for a total response rate of 32%. For surgeons, our study prompt was highly correlated with SAQ scores for assessment of teamwork and general working conditions (0.82, 95% confidence interval [CI], 0.57–1.93), whereas a moderate correlation was seen for anesthesiologists (0.59; 95% CI, 0.25–0.78). The correlation between the study prompt and SAQ scores for working climate was 0.91 (95% CI, 0.77–6.39) for surgeons and 0.50 (95% CI, 0.13–0.74) for anesthesiologists. The response rate among scrub nurses and circulating nurses was 20% (n = 6) and 40% (n = 12), respectively, yet when the 2 categories of nurse are combined, their response rate parallels that of surgeons (27.7% vs 29.2%, respectively). Nonetheless, the response rate among nurses showed poor correlation with wide CIs, prohibiting meaningful inferences.

## DISCUSSION

The response rates for dynamic surveys are low when sent by email, which we hope to mitigate with in-OR devices. Focused interviews with our nursing teams did not identify any systematic barriers to a higher completion rate. Our study demonstrates that a single prompt with a Likert scale response system is well correlated with the SAQ scores in the domains of teamwork and working conditions for surgeons and anesthesiologists. Ease of access and simplicity of novel devices may facilitate higher response rates and more accurate assessments of OR team satisfaction for all team members.
